# 795 A 12-Year Experience in Lower Extremity Acute Burns Reconstruction

**DOI:** 10.1093/jbcr/irae036.336

**Published:** 2024-04-17

**Authors:** Jose Antonio Arellano, Tiffany Jeong, Mario Alessandri-Bonetti, Hilary Liu, Guy M Stofman, Francesco Egro

**Affiliations:** University of Pittsburgh Medical Center, Pittsburgh, PA; University of Pittsburgh Medical Center, Pittsburgh, PA; University of Pittsburgh Medical Center, Pittsburgh, PA; University of Pittsburgh Medical Center, Pittsburgh, PA; University of Pittsburgh Medical Center, Pittsburgh, PA; University of Pittsburgh Medical Center, Pittsburgh, PA

## Abstract

**Introduction:**

A paucity of studies investigated the outcomes of flap reconstruction in lower extremity acute burn. The aim of this study is to report outcomes of lower extremity acute burn needing pedicled or free flap coverage.

**Methods:**

We conducted a retrospective study to review patients with lower extremity acute burns needing either pedicled or free muscle flap coverage between August 2010 and December 2022 at an ABA-certified burn center. Gathered data included demographics, comorbidities, type of injury, total body surface area involved, anatomical location, indication for flap coverage, timing of reconstruction, follow-up, number and type of re-operations, and complications. Chi-square test was used to measure differences in complication rate between pedicled and free flaps.

**Results:**

Over a 12-year period, 31 patients underwent 38 muscle flaps for lower extremity acute burn and were included in the study. In total, 81% were males and 19% females, with a mean age of 47±12 years and a mean BMI of 28.4±7.2. Thermal injury was the most common cause of soft tissue defects (92%), while electrocution caused the remaining 8%. Most common injured area was the lower leg (82%). Mean time between the day of injury and reconstructive surgery was 21.3±9.5 days and the mean follow up period was 11.2±11.8 months.

Out of 38 flaps, 28 were pedicled muscle flaps (14 gastrocnemius flaps, 12 soleus flaps, 1 anterior tibial muscle flap, 1 abductor digiti minimi muscle flap) and 10 were free muscle flaps (8 rectus abdominis flaps, 1 vastus lateralis flap, 1 latissimus dorsi flap).

In the free flap group complications were: total flap loss (20%), wound healing issues (30%), hematoma (20%), infection (10%). In the pedicled flap group complications were wound healing issues (14.3%), skin graft loss (10.7%), tip necrosis (7.1%) and infection (3.6%).

Free flaps showed a significantly higher rate of need for re-operation (50% vs 35.7%, p=0.048) and total flap loss (20% vs 0%, p=0.015) compared to pedicled flaps.

**Conclusions:**

Flap coverage in lower extremity acute burns is rarely employed. Yet, in the case of critical structure exposure, it is often necessary. However, it is important to be aware of the high risk of complications, especially for more complex reconstructions needing a free tissue transfer.

**Applicability of Research to Practice:**

Surgeons and patients will benefit from a greater awareness of the high risk of complications seen in free tissue transfer during lower extremity acute burn reconstruction.

